# Recycling Compatible Organic Electrode Materials Containing Amide Bonds for Use in Rechargeable Batteries

**DOI:** 10.3390/polym15224395

**Published:** 2023-11-13

**Authors:** Masaru Yao, Hikaru Sano, Hisanori Ando

**Affiliations:** Research Institute of Electrochemical Energy, Department of Energy and Environment, National Institute of Advanced Industrial Science and Technology (AIST), 1-8-31 Midorigaoka, Ikeda 563-8577, Japan; hikaru.sano@aist.go.jp (H.S.); h-ando@aist.go.jp (H.A.)

**Keywords:** organic battery, recycling, long cycle life, amide bond, oligomer, quantum chemistry calculation

## Abstract

Organic rechargeable batteries that do not use any scarce heavy metals are candidates for the next generation of rechargeable batteries; although, it is not easy to realize both high capacity and long cycle life. Organic compounds linked by amide bonds are expected to have superior recycling properties after battery degradation, since they will become a single monomer upon hydrolysis. In this study, anthraquinone was chosen as a model redox active unit, and dimeric and trimeric compounds were synthesized, their cycle performances as electrode materials for use in rechargeable batteries were compared, and a trend in which oligomerization improves cycle properties was confirmed. Furthermore, quantum chemistry calculations suggest that oligomerization decreases solubility, which would support a longer life for oligomerized compounds. This methodology will lead to the development of organic rechargeable batteries with further environmental benefits.

## 1. Introduction

In order to realize a carbon-neutral society, many industries are working to reduce greenhouse gas emissions, and the electrification of apparatus currently powered by fossil fuels is indispensable. For example, electric vehicles are becoming increasingly widespread, and in recent years, the electrification of ships and aircraft has been considered. One of the most important devises in their development is a rechargeable battery, and the use of rechargeable lithium batteries is expected. Requirements for this battery system have conventionally focused on energy density and cycle life; however, in recent years, recyclability has become increasingly important in addition to the reduction of environmental burden related to minor heavy metal use and the improvement in thermal safety. To meet these requirements, organic batteries are one of the possible candidates [1,2,3,4,5,6,7,8,9]. In addition to being free of minor heavy metals, organic batteries offer high capacity and could contribute to thermal safety improvement [10]. Among the reported active materials, the number of the examples of low-molecular-weight materials that can realize high capacity is increasing. So far, we have also reported many organic active materials, mainly low-molecular-weight materials [11]. However, in many cases, small molecules do not have sufficient cycle life characteristics, and it is a challenge to balance high capacity and good cycle characteristics.

One of the reasons for insufficient life properties is the dissolution of low-molecular-weight active materials into the electrolyte solutions. To this issue, polymeric or oligomeric compounds with covalently bonded redox centers have been reported to extend the life of a battery [12,13,14,15,16,17]. On the other hand, when the bonding mode is excessively strong, it becomes too stable, and this high stability is not always preferable considering the battery recycling process.

**Figure 1:**
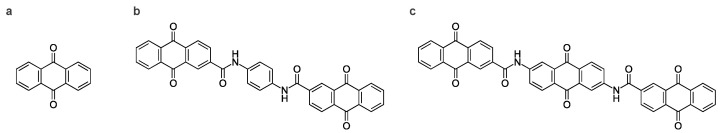
Chemical structures of (**a**) anthraquinone (AQ), (**b**) AQ amide dimer, and (**c**) AQ amide trimer.

In this study, we focused on the amide bond as a linkage system that is easy to use and effective in the recycling process. An amide bond is a naturally existing bonding mode and is known to be biodegradable [18,19]. This bond formally consists of a dehydration condensation of a carboxy group with an amino group and it is often synthesized with the reaction of an acid halide with an amino group. Redox-active polymers composed of such linkage systems can be converted to monomers via hydrolysis; thus, even if active materials or other components are degraded during battery operation, they can be returned to their raw materials in a subsequent recycling process. In this study, anthraquinone was selected as a model redox center, and its oligomers with amide bonds were synthesized to investigate the applicability of the amide oligomers as active materials (Figure 1).

## 2. Materials and Methods

### 2.1. Materials

A monomer, anthraquinone (Kanto Chemical Co., Inc., Tokyo, Japan), binding units of the oligomers, 2,6-diaminoanthraquinone (Tokyo Chemical Industry Co., Ltd.: TCI, Tokyo, Japan), anthraquinone-2-carbonyl chloride (TCI), and diaminobenzene were each used as purchased. Other common synthetic reagents were also purchased and used as is. The synthesized compounds were characterized by infrared (IR) (Frontier FT-IR spectrometer, PerkinElmer, Inc., Waltham, MA, USA) equipped with an attenuated total reflection (ATR) stage, mass spectrometry (ACQUITY SQD mass spectrometer, Waters, Milford, MA, USA) equipped with an atmospheric solids analysis (ASAP) probe, melting point measurement (OptiMelt MPA-100, Stanford Research Systems, Sunnyvale, CA, USA), scanning electron microscope (SEM) (JSM-IT100, JEOL Ltd., Akishima, Japan) with an energy dispersive X-ray analysis (EDX) probe (DrySD™25, JEOL Ltd., Akishima, Japan), and X-ray photoelectron spectroscopy (XPS) measurement (PHI 5000 VersaProbe, ULVAC-PHI, Chigasaki, Japan).

### 2.2. Synthesis

#### 2.2.1. Anthraquinone Amide Dimer

*p*-Phenylenediamine (24 mg, 0.2 mmol) was dissolved in a mixture of dehydrated *N*,*N*-diisopropylamine (1 mL) and toluene (2 mL) and mixed with a dry *N*,*N*-dimethylformamide (DMF) solution (2 mL) of anthraquinone-2-carbonyl chloride (108 mg, 0.4 mmol). The resulting solution was refluxed at 110 °C for 4 h. After slow cooling, the precipitate was filtered off and washed to afford 27 mg of the yellow target product. The compound was insoluble in most solvents. Yield: 23%; MP: >400 °C; ASAP-MS (*m/z*): calcd. for C_36_H_20_N_2_O_6_: 576; found: 577 [M+H]^+^; IR (cm^−1^): 3292 (NH stretch), 1674, 1650 (C=O stretch) 1591 (aromatic ring stretch), 1548 (amide NH bend); XPS (eV): 284.8 (C-C, C-N, C-H) (calibration reference), 287.2 (C=O), 288.0 (N-C=O), 290.4 (π-π*).

#### 2.2.2. Anthraquinone Amide Trimer

2,6-Diaminoanthraquinone (120 mg, 0.5 mmol) was dispersed in a mixture of dehydrated *N*,*N*-diisopropylamine (2.5 mL) and xylene (5 mL), and to this dispersion anthraquinone-2-carbonyl chloride (274 mg, 1 mmol) in dehydrated DMF solution (5 mL) at 0 °C was added. The resulting solution was heated overnight using an oil bath set at 140 °C. After allowing the mixture to cool slowly, the precipitate was removed via filtration and washed to give 24 mg of the light brown target compound. The compound was also insoluble in most solvents. Yield: 7%; MP: >400 °C; ASAP-MS (*m/z*): calcd. for C_44_H_22_N_2_O_8_: 706; found: 706 [M]^+^; IR (cm^−1^): 3240 (NH stretch), 1669, 1649 (C=O stretch), 1588, 1578 (aromatic ring stretch), 1518 (amide NH bend); XPS (eV): 284.8 (C-C, C-N, C-H) (calibration reference), 287.3 (C=O), 288.1 (N-C=O), 290.4 (π-π*).

### 2.3. Battey Measurements

The property of the prepared materials as a positive electrode material was examined using coin-type lithium metal half cells. First, a positive electrode containing a synthesized material was prepared as follows. An organic active material powder, acetylene black as the conductive additive, and polytetrafluoroethylene as the binder were prepared, mixed in the weight ratio 4:5:1 in a mortar to prepare a composite sheet. The sheet was then pressed onto a mesh-type stainless steel (SUS 316L) current collector. The amount of active material was 1.5 or 3.0 mg per electrode. Next, an IEC R2032 coin-type cell was assembled with the prepared positive electrode, lithium metal negative electrode, separator, and the electrolyte solution of lithium bis(trifluoromethanesulfonyl)imide (LiTFSI)/diglyme (1 mol/L) (0.2 mL). As a preliminary electrochemical measurement, cyclic voltammetry (CV) was applied to the above-mentioned coin cells using an electrochemical analyzer (SI 1280B, Solartron Analytical, Leicester, UK) with a scan speed of 0.1 mV/s in a voltage range of open-circuit voltage to 1.5 V vs. Li_C.E._ at room temperature. In the battery test, the prepared coin-type cells were galvanostatically discharged and charged at a current density of 20 or 40 mA/g (followed by 20 mA/g) with a voltage range of 1.5–3.0 V vs. Li_C.E._ at 30° C using a battery evaluation system (BLS series, Keisokuki Center Co., Ltd., Osaka, Japan and ABE system, Electrofield Co., Ltd., Osaka, Japan).

### 2.4. Hydrolysis Test

A small amount of anthraquinone amide trimer was dispersed in a mixture of sulfuric acid aqueous solution/1,4-dioxane and the dispersion was heated using an oil bath set at 110 °C and stirred overnight. After allowing the mixture to cool to room temperature, the dispersion was poured into water and the precipitate was collected. The filtrate was then basified with KOH to precipitate organic salts. The obtained reactants were analyzed without purification. ASAP-MS (*m/z*): calcd. for diamino anthraquinone (AQ-(NH_2_)_2_) C_14_H_10_N_2_O_2_: 238, carboxy anthraquinone (AQ-COOH) C_15_H_8_O_4_: 252, found: 239 [M_AQ-(NH2)2_+H]^+^, 253 [M_AQ-COOH_+H]^+^.

### 2.5. Theoretical Calculations

To estimate the intermolecular interaction of monomer or oligomers, the density functional theory-based optimization was first performed using a popular basis set of 6-31G(d) and a functional of BLYP [20,21] with Grimme’s D3 dispersion correction [22]. For the actual calculation of the intermolecular interaction, a single point calculation at 6-311G(d)/B3LYP-D3 level was used [22,23]. To take the solvation effect into consideration, the polarizable continuum model (PCM) specifically with the solute electron density model (SMD) [24] was applied to a series of molecules. A single point calculation in an ether was conducted for their optimized structures at the same level. In these calculations, the GAUSSIAN 16 program package [25] was used and the calculated molecules were visualized with Gauss View 6.1.1 [26].

## 3. Results

### 3.1. Synthesis

**Figure 2:**
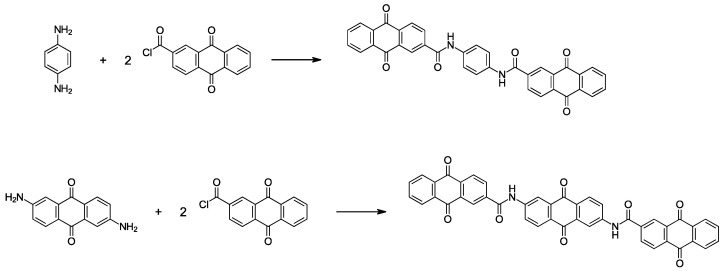
Synthesis routes for AQ amide dimer and AQ amide trimer.

An AQ amide dimer was synthesized via condensation of an AQ carbonyl chloride with a diaminobenzene, and an AQ amide trimer was prepared via condensation of the carbonyl chloride compound with a diamino-AQ (Figure 2). These newly synthesized oligomers are virtually insoluble in ordinary organic solvents at room temperature. This insolubility is important for the long cycle life of the active materials. However, this low solubility makes it difficult to apply normal solution-based purification techniques. Here, to determine whether the desired reaction had proceeded, we used other techniques that are suited to the analysis of solids: MS (mass spectra) indicted their molecular weights (Figure S1), IR measurement (Figure S2) confirmed that the samples had bands derived from the amide bonds, EDX measurement (Figure S3) analyzed their composition, and XPS provided the information on the bonding state of carbon atoms from their C_1s_ spectra (Figure S4). Here we describe our interpretation of the results of the XPS measurements, which may not be so common in the analysis of organic materials. The C_1s_ XPS peaks obtained for the synthesized amide dimer and amide trimer were divided into four peaks (Figure S4). The decomposed XPS peaks are assigned, in order from low energy to high energy, to the major C-C bonds (plus C-N and C-H) at 285 eV, the C=O bonds of the quinone skeletons at 287 eV, the N-C=O bonds for the amide moieties at 288 eV, and the broad π-π* shake-up at 290 eV, which are commonly seen in aromatic compounds. In both the amide dimer and trimer, the N-C=O peaks attributed to the amide bond were identified, complementing the amide bond absorption observed in the IR measurement. Furthermore, comparing the molecular structures of the dimer and trimer, both have the same two amide bonds, whereas the number of carbonyls in the quinone is four in the dimer and six in the trimer. In actual analysis of the obtained spectra, it is clear that the quinone C=O area around 287 eV compared to the peak attributed to N-C=O at around 288 eV is larger in the trimer than in the dimer, which is consistent with the target structure. In addition, SEM measurements (Figure S5) suggested that the obtained samples have a needle-like crystalline state. Although the resulting powder may contain impurities, these analytical measurements support that the synthesized compounds have the desired structure, which contains anthraquinone structures connected by amide bonds.

### 3.2. Electrochemical Tests

**Figure 3:**
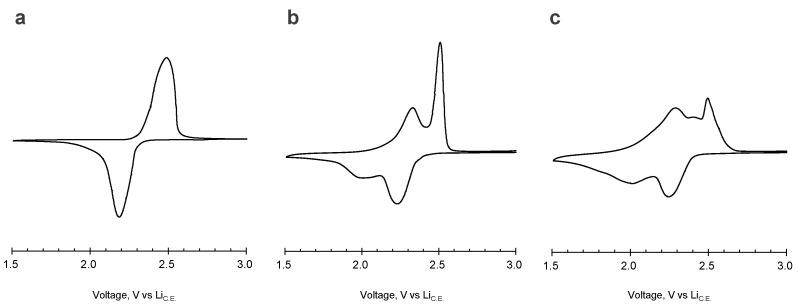
Cyclic voltammograms (second cycle) of the electrodes using (**a**) AQ, (**b**) AQ amide dimer, and (**c**) AQ amide trimer (scan rate: 0.1 mV/s, temperature: r.t.).

Figure 3 shows the cyclic voltammograms of the prepared electrodes. The AQ electrode has one cathodic wave at 2.2 V vs. Li_C.E._, and one anodic wave at around 2.5 V vs. Li_C.E._ On the other hand, the synthesized AQ amide dimer and AQ amide trimer showed different behaviors compared to the monomer, although the voltammograms of the two oligomers are similar each other. The dimer has two cathodic waves around at 2.2 and 2.0 V vs. Li_C.E._, followed by anodic peaks around at 2.3 and 2.5 V vs. Li_C.E._ The trimer also has two cathodic waves and two anodic waves at almost the same voltage positions (cathodic: 2.2 and 2.0 V vs. Li_C.E._; anodic: 2.3 and 2.5 V vs. Li_C.E._). The splitting and broadening of the redox waves are considered to originate from intramolecular interactions caused by the oligomerization.

**Figure 4:**
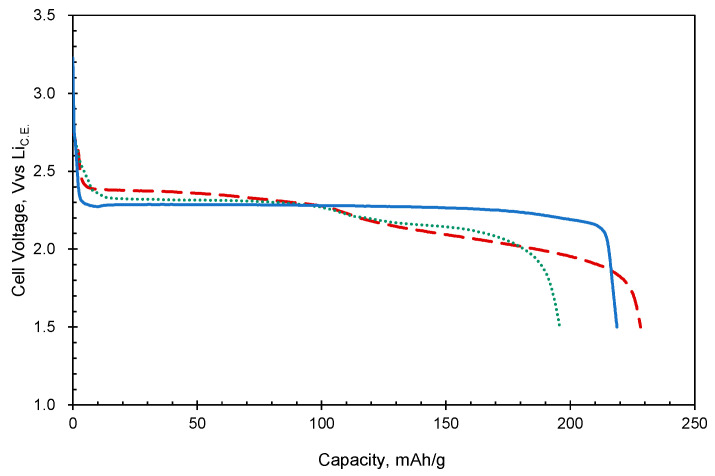
Initial discharge curves of the electrode (active material content: 3 mg/electrode) using AQ (solid blue line), AQ amide dimer (dotted green line), and AQ amide trimer (dashed red line) at the current density of 20 mA/g.

Figure 4 shows the initial discharge curves of the AQ monomer, amide dimer, and amide trimer. In the case of the AQ monomer, the capacity obtained in the initial discharge was 219 mAh/g, which is close to the theoretical capacity (258 mAh/g) [17]. Then, for the dimer and trimer, the capacities obtained in the initial discharge process were 196 and 225 mAh/g, respectively, which are close to the theoretical capacities of 186 and 227 mAh/g, where two electrons per the AQ moiety are assumed to be transferred. (Although it is not clear why the dimer capacity exceeds the theoretical value, it may be due to impurities.) The AQ electrode has one voltage plateau region around at 2.3 vs. Li_C.E_. On the other hand, the oligomers have two plateau regions: 2.3 and 2.1 V vs. Li_C.E._ for the dimer, and 2.4 and 2.0 V vs. Li_C.E._ for the trimer. This behavior indicates a stepwise electron transfer reaction for these oligomers. These values are close to the cathodic peak positions observed in the CV measurement; this probably reflects the electronic effects of the introduced electron-donating NH moieties and electron-withdrawing C=O groups in the amide bond. Going back to the utilization ratio, the observed high values are likely due to the effective intermolecular contact of the AQ moiety, a redox center, and the exhibiting of some degree of electronic conduction. We previously reported similarly high utilization for oligomers linked by triple bonds, in which we considered that the triple bonds could also contribute to intramolecular conduction since the volume occupied by the triple bond is small and a delocalized structure via the triple bond can be drawn. On the other hand, for the present molecule, intramolecular electronic conduction through the amide bond, where the electrons are localized, is not expected. Therefore, intermolecular interactions are considered to be dominant.

**Figure 5:**
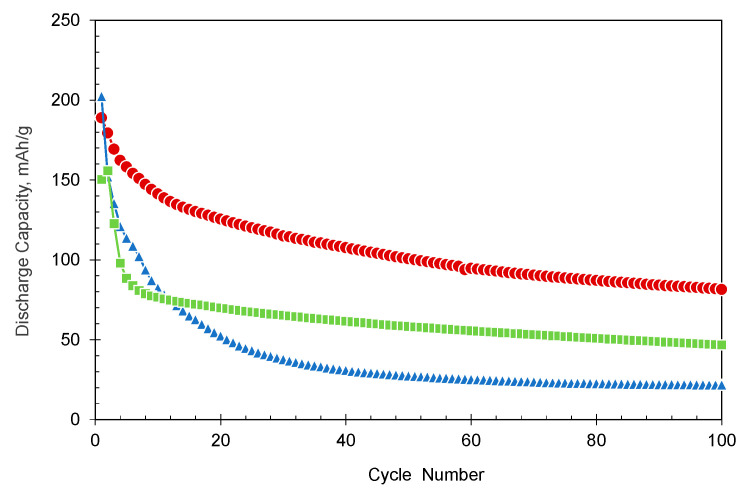
Cycle life performance of the electrode using AQ (▵), AQ amide dimer (□), and AQ amide trimer (○). Here, electrodes containing 1.5 mg of the active materials were used. The capacity values are expressed as the sum of the value obtained at a current density of 40 mA/g and the value gained at a subsequent current density of 20 mA/g.

Next, the effect of oligomerization on cycle properties was examined. Figure 5 compares the actual cycle properties obtained for the AQ monomer and oligomers. (Charge/discharge curves during the cycle test are shown in Figure S6). As previously reported, the capacity of the monomer significantly decreases along with cycling. Although multiple factors can be considered for the degradation, a dissolution can be regarded as at least one clear factor, since the coloring of the electrolyte was observed when the battery was disassembled after a few cycles. To reduce the solubility, we attempted oligomerization with amide bonding in this study. As for the dimer, the capacity after 100 cycles was 47 mAh/g, showing higher capacity retention characteristics than 22 mAh/g for the AQ monomer. This is likely due to a certain decrease in solubility in the electrolyte via dimerization. Furthermore, the capacity of the trimer after 100 cycles was 82 mAh/g, showing better capacity retention properties than the monomer and dimer. This trend is similar to that of the AQ oligomers linked by triple bonds in our previous paper [17], while the triple bond seems to be more effective than the amide bond. That being said, it was confirmed once again that oligomerization via amide bonds is also useful for cycle improvement.

**Figure 6:**
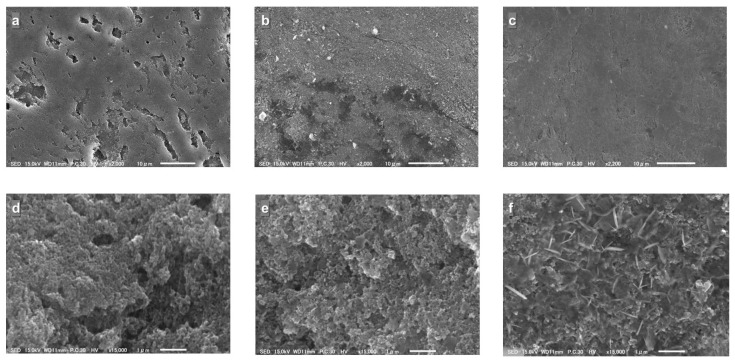
SEM images of the electrodes after 100 cycles. The surface images (**a**–**c**) and the cross sectional ones (**d**–**f**). ((**a**,**d**) AQ; (**b**,**e**) AQ amide dimer; (**c**,**f**) AQ amide trimer).

To find the cause of the deteriorations, the cells were dissembled after 100 cycles and the state of their internal components was examined. After the cycle test, some separator coloring was observed for all the cells (Figure S7), suggesting the dissolution of the active materials as described above. While the color changes for the separators for the AQ monomer and dimer are similar, the degree of change for the trimer was milder than that for the monomer and dimer. (It should be noted here that the amount of dissolution cannot be determined only by comparing the degree of coloration of the separator, because the color tone of the active material itself varies from sample to sample: AQ: pale yellow; AQ amino dimer; yellow; AQ amino trimer: light brown). Next, an SEM image comparison of the electrodes after cycling is shown in Figure 6. Comparing the surface images (Figure 6a–c) of the three cycled electrodes, the surface smoothness appears to increase in the order of the monomer, dimer, and trimer. Since these electrode surfaces are originally smooth, this observation indicates that the surface has been roughened during cycling. Figure 6d–f shows enlarged cross sectional images of the electrodes. While there is no clear difference between the electrodes of the monomer and dimer, the trimer electrode clearly retains a needle-like powder morphology that is likely to be derived from the pristine trimer powder. This observation is thought to also indicate that of the three active materials, the trimer had the lowest solubility into the electrolyte solution during cycling.

### 3.3. Recycling Ability Test: Hydrolysis Reaction

**Figure 7:**
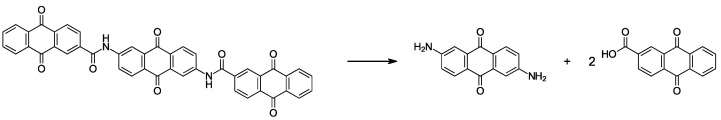
Hydrolysis reaction for the AQ amide trimer.

As explained in the introduction, amide bonds are known to undergo hydrolysis reactions. Therefore, the synthesized oligomers should revert to small soluble fragments after the hydrolysis reaction. To verify this concept, we attempted a preliminary hydrolysis reaction for the prepared amide trimer. The expected reaction is described below (Figure 7).

**Figure 8:**
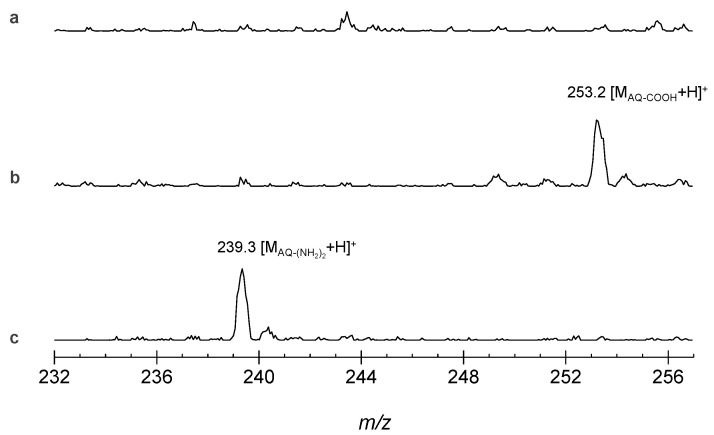
Mass spectral change via hydrolysis reaction for the AQ amide trimer. (**a**) Pristine powder, (**b**) precipitate obtained from an acidic solution, and (**c**) precipitate taken from a basic condition.

The mass spectral charts of the material before (Figure 8a) and after hydrolysis (Figure 8b,c) are shown below. In the state before hydrolysis, there is no noticeable signal in the measurement region, whereas after hydrolysis, a peak appears at *m/z* = 253 for the precipitate obtained from an acidic aqueous solution. By adjusting the pH of the solution from acidic to basic, another precipitate was obtained, which shows an additional peak at *m/z* = 239. The former corresponds to the protonated mass of the carboxyanthraquinone and the latter to the protonated mass of the diaminoanthraquinone. (In acidic solutions, amino compounds, R-NH_2_, remain dissolved because they are protonated to R-NH_3_^+^ and do not precipitate without neutralization treatment. Conversely, carboxy compounds, R-COOH, precipitate under acidic conditions, and turn into R-COO^−^ under basic conditions, thus dissolving. The present results reflect such characteristics of amino and carboxy groups.) Hence, this observation indicates that the desired hydrolysis reaction proceeded and the insoluble amide AQ trimer became smaller fragments. Although the reaction conditions have not been optimized in this case, the yield and other factors could be improved by adjusting the type and amount of catalyst, reaction temperature and concentration, and with the use of special enzymes.

### 3.4. Quantum Chemistry Calculation

**Figure 9:**
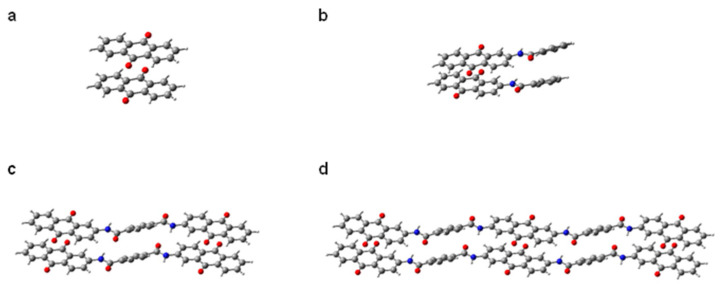
Optimized stacking motives for (**a**) AQ, (**b**) AQ-amide monomer, (**c**) AQ amide dimer model, and (**d**) AQ amide trimer model. Color coding: grey, carbon; white, hydrogen; red, oxygen; blue, nitrogen.

**Figure 10:**
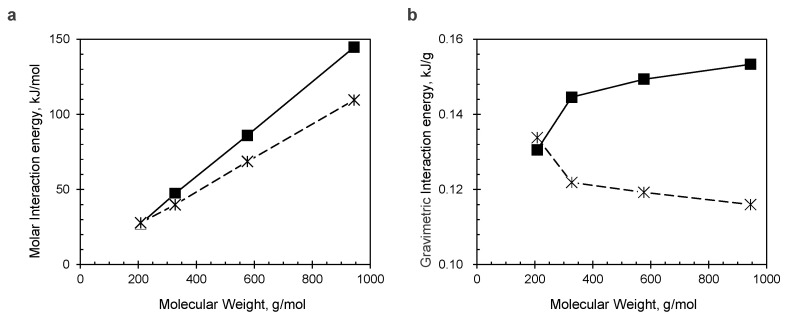
Comparison of the intermolecular attractive energy (■; solid line) and solvation energy (*; dashed line) calculated for the AQ model compounds. (**a**) Molar interaction energy vs. molecular weight and (**b**) energy per molecular weight (i.e., gravimetric value) vs. molecular weight.

To theoretically address the effect of oligomerization on the cycle performance described above, quantum chemical calculations were performed. First, we estimated how the intermolecular interactions in the crystal increase with dimerization and trimerization. To simplify the model, calculations were performed for a total of four types of molecules: the AQ monomer, a monomer with AQ linked to a phenyl group by an amide bond, a dimer with one benzene ring linked to two AQs by two amide bonds, and a trimer with two benzene rings linked to three AQs in a linear fashion by four amide bonds (Figure 9). All the AQ-based molecules were calculated to have a stacked structure with about 3.3–3.4 Å interplane distance, which is close to the interlayer distance of graphite (3.35 Å). Also, these compounds show a slightly displaced stack motif as the optimal solutions. The observed offset π-stacking is typical for π-conjugated polycyclic molecules. The intermolecular coupling energies were calculated to be 27.2, 47.3, 86.0, and 144.8 kJ/mol for the original AQ monomer, amide monomer, amide dimer, and amide trimer, respectively (Figure 10a). Since the van der Waals force, which is regarded as a weak attractive interaction, is less than 5 kJ/mol, hydrogen bonding, which is comparatively strong, is about 10–40 kJ/mol, and covalent bonding is about 100–300 kJ/mol; the calculated value for the trimer is very strong as an intermolecular force.

In addition, the solvation energies of these oligomers in ether were also calculated. Values of 27.8, 39.9, 68.7, and 109.5 kJ/mol were obtained for the AQ monomer, amide-monomer, dimer, and trimer, respectively, which also show an upward trend with increasing molecular weight (Figure 10a). However, the increase rate is different: the intermolecular energy shows a larger increase rate than the solvation one. To advance this discussion, we examined the behavior of the obtained intermolecular interaction stabilization energies and solvation energies normalized by molecular weight (Figure 10b). Interestingly, the intermolecular interactions and solvation show opposite behavior: the former increases in value with increasing molecular weight, while the latter decreases. In other words, the calculations indicate that as the number of the repeating unit of these oligomers increases, they become more insoluble in the solvent.

## 4. Discussion

**Figure 11:**
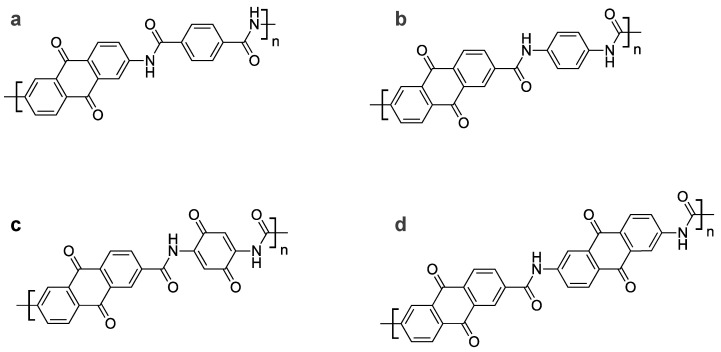
Some hypothetical amide polymers. (**a**,**b**) AQ polymers containing phenylene moieties; (**c**) AQ-BQ type polymer; (**d**) polymer composed of only AQ and amide units.

With regard to organic rechargeable batteries, there have been a number of reports showing that a multimerization of redox centers leads to an improvement in cycle performance [12,13,14,15,16,17]. Although there have been some cases in which polymerization of some redox centers has led to a decrease in capacity, in the case of AQs, so far there have not been many reports of polymerization leading to a decrease in capacity. To date, polymerization/oligomerization of AQ has been reported such as those connected by sulfur atoms [12] and those connected by triple bonds [17]. Although these compounds are extremely insoluble in solvents, which improves cycle life, their insolubility becomes an obstacle to recycling when battery performance deteriorates. The amide bond that we focused on in this study undergoes a hydrolysis reaction. Even if the battery is degraded, the contained polymer can be easily converted back to the original monomers, which could lead to good recycling properties. In this study, we used AQ as a model compound and linked the moieties with amide bonds. The results showed that the dimer form of AQ exhibited slightly better cycling properties than the AQ monomer, and the trimer form showed a more improved effect. Furthermore, we confirmed that an amide-based oligomer undergoes a hydrolysis reaction to yield its fragment monomers. Quantum chemistry calculations also suggest that oligomerization is effective in reducing solubility. Although we have only been able to synthesize up to a trimer, it is inferred that further polymerization to higher molecular weights will improve performance even further. For example, if the following polymers described in Figure 11a,b could be synthesized, they could be a long-life material with a theoretical capacity of 146 mAh/g. The introduction of an electron-donating group such as an amino group into the AQ structure leads to a decrease in voltage; on the contrary, an adjacent electron withdrawing C=O group is likely to result in a higher voltage, which would be effective in improving the energy density.

Moreover, the benzene ring moiety used for bonding still leaves some places where substituents can be introduced. For example, as shown in Figure 11c, assuming that the bonding moiety is a benzoquinone structure, the theoretical capacity would be 269 mAh/g. In addition, if the structure of our synthesized trimer is extended to a polymer, Figure 11d, the theoretical capacity would be 215 mAh/g. These designs will improve the energy density.

## 5. Conclusions

Concerning multimerization, which is effective in extending the life of active materials in rechargeable organic batteries, the use of amide bonds was investigated in this study. With a focus on anthraquinone (AQ) as a model redox center, its amide dimer and trimer were synthesized with a condensation reaction of an anthraquinone carbonyl chloride with *p*-phenylenediamine and with a diamino-anthraquinone. In the battery test, the synthesized compounds and AQ showed similar initial discharge capacities, approximately 200 mAh/g, whereas there was a difference in their cycle performance, indicating that oligomerization using amide bonds has a favorable effect on the cycle properties. Quantum chemistry calculations gave quantitative values for the intermolecular attractive interaction and solvation energy, predicting that oligomerization would reduce the solubility of the active material.

Reports that oligomerization and polymerization are effective in improving the cycle properties of organic active materials have been increasing recently, and it can be considered that the main factor is the reduction of solubility level via increasing the repeating number of redox units in each molecule. On the other hand, such a strong bond will make the recycling process difficult after battery deterioration. Among many kinds of chemical bonds, amide bonds, although physically strong, are known to decompose chemically upon hydrolysis. Also, an artificial technique for dissolving these bonds has been established. Hence, even after degradation of the battery, the multimerized molecules can be decomposed to the monomer level through a hydrolysis process. The constituent units can be easily extracted and used in the subsequent repolymerization reaction. Although preliminary, this concept was verified with the confirmation that a synthesized amide oligomer (specifically, amide trimer) undergoes hydrolysis and reverts to its monomer form. We expect that higher capacity redox centers will lead to the development of high-energy, organic rechargeable batteries with less environmental burden.

## Data Availability

Data are available upon request.

## Supplementary Materials

The following supporting information can be downloaded at: https://www.mdpi.com/article/10.3390/polym15224395/s1, Figure S1: Mass spectra; Figure S2: IR spectra; Figure S3: EDX spectra; Figure S4: XPS spectra; Figure S5: SEM images of the samples; Figure S6: Charge/discharge curves during cycling; Figure S7: Photograph of the separators after cycling.
