# Supplementary material for: Recycling Compatible Organic Electrode Materials Containing Amide Bonds for Use in Rechargeable Batteries
Source: Polymers (Basel). 2023 Nov 13;15(22):4395. doi: 10.3390/polym15224395 (PMC10675302; doi:10.3390/polym15224395)
Supplement: Supplementary file 1 [file polymers-15-04395-s001.zip › polymers-2651513-supplementary.pdf]

Supporting Information

Recycling Compatible Organic Electrode Materials Containing Amide Bonds for Use in Rechargeable Batteries

Masaru Yao*, Hikaru Sano, and Hisanori Ando

Research Institute of Electrochemical Energy, Department of Energy and Environment, National Institute of Advanced Industrial Science and Technology (AIST), 1-8-31 Midorigaoka, Ikeda, Osaka 563-8577, Japan

* Correspondence: m.yao@aist.go.jp

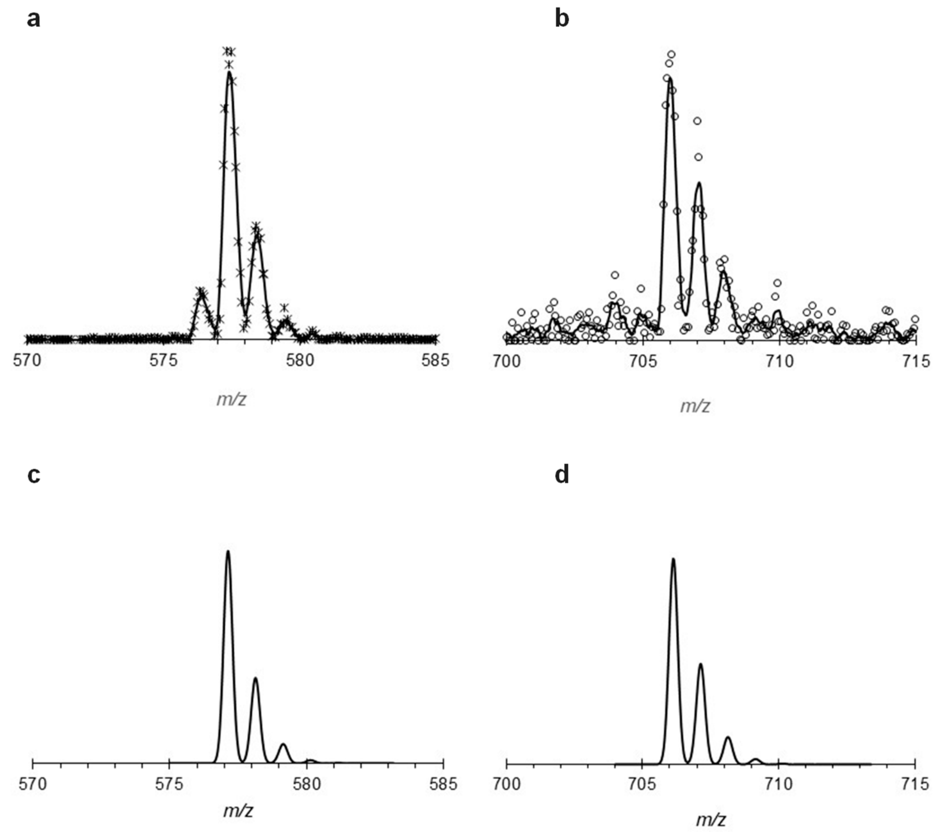

Figure S1. Mass spectra of the (a) amide-bonded dimer and (b) amide-bonded trimer along with (c,d) their simulated patterns considering the isotope abundance ratios.

a

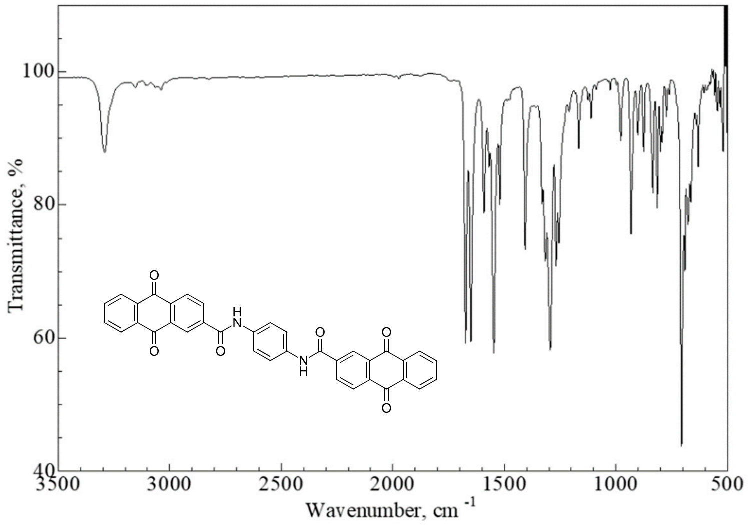

b

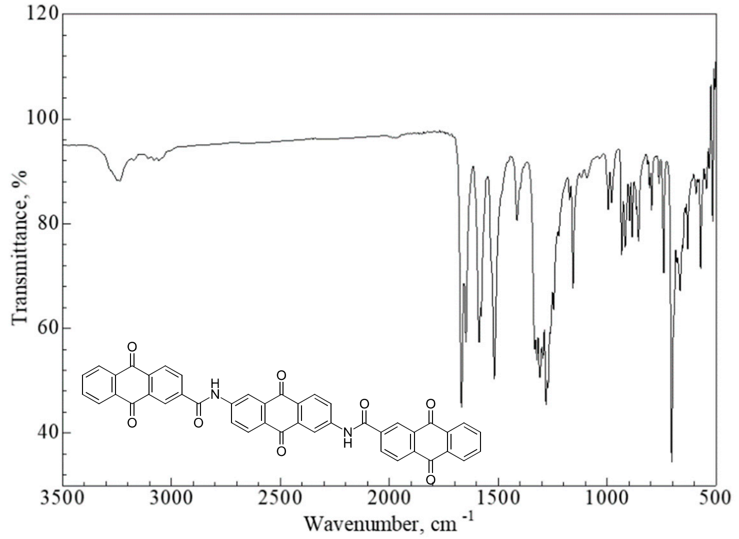

Figure S2. IR spectra of the (a) amide-bonded dimer and (b)amide-bonded trimer.

a

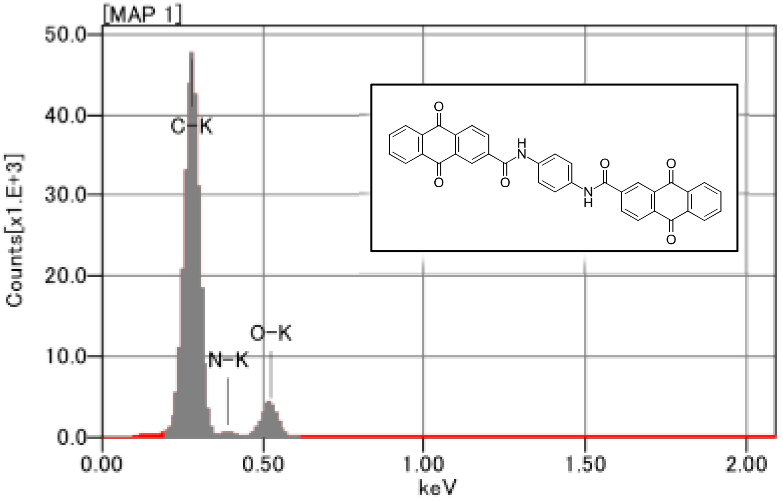

Atomic Species	mass%	atm%	σ
C	70.82	75.50	0.01
N	10.00	9.15	0.06
O	19.18	15.35	0.04
Sum	100.00	100.00	

b

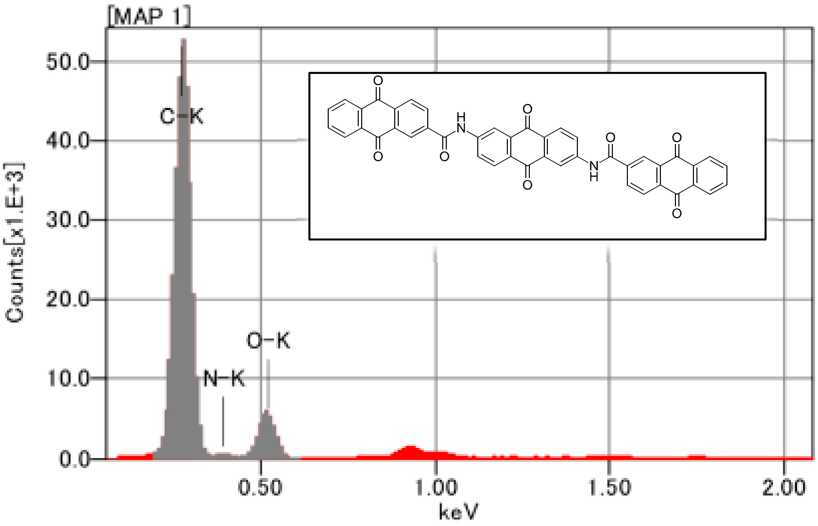

Atomic Species	mass%	atm%	σ
C	69.26	74.24	0.01
N	8.89	8.18	0.06
O	21.85	17.58	0.04
Sum	100.00	100.00	

Figure S3. EDX spectra for (a) the amide-bonded dimer and (b) amide-bonded trimer.

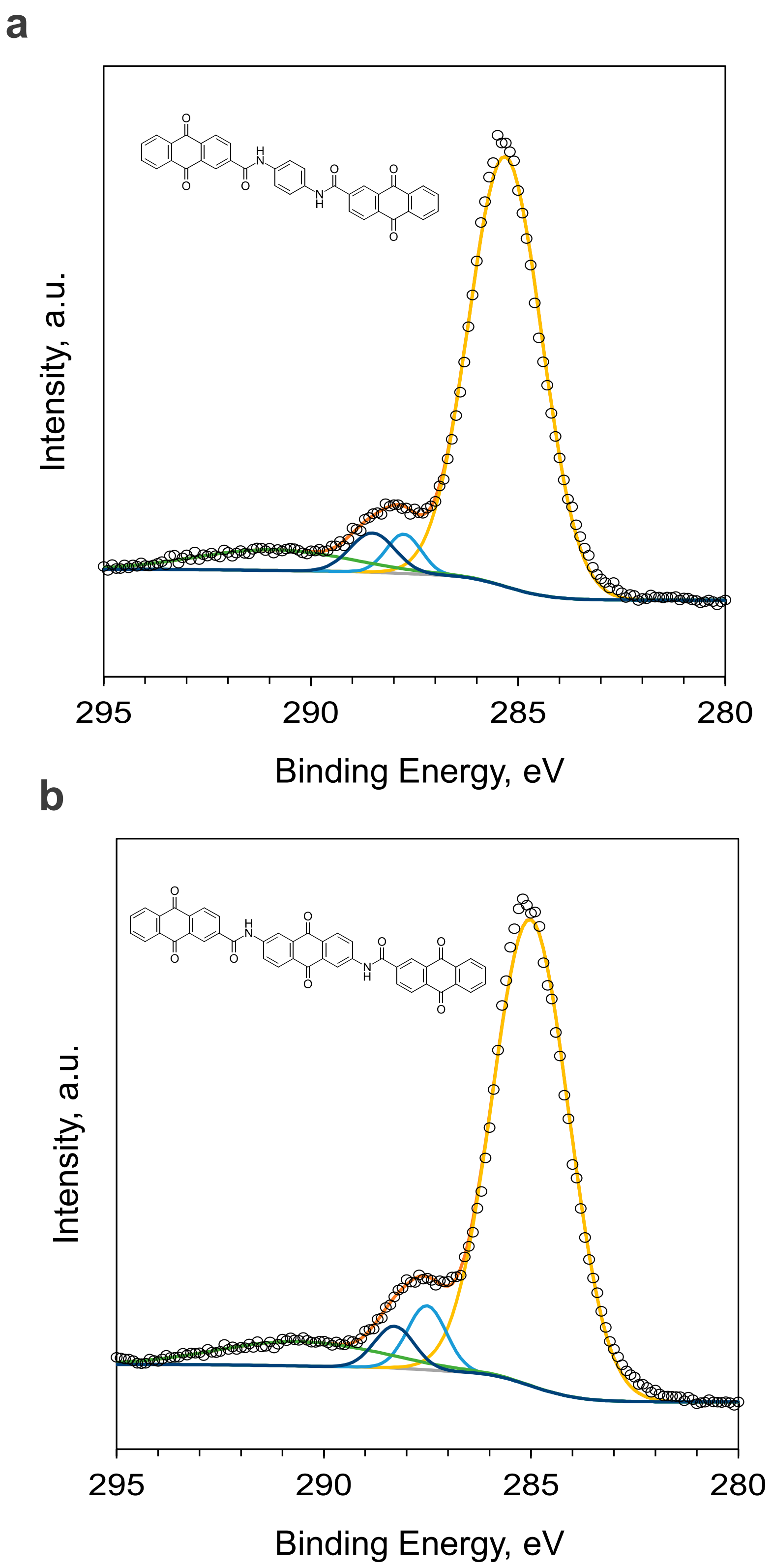

Figure S4. XPS spectra of the (a) amide-bonded dimer and (b)amide-bonded trimer. Monochromatic Al Kα (1.4866 keV) was used. These C_{1s} profiles were decomposed into four peaks and Shirley's background by the curve-fitting software XPSPEAKS 4.1.

a

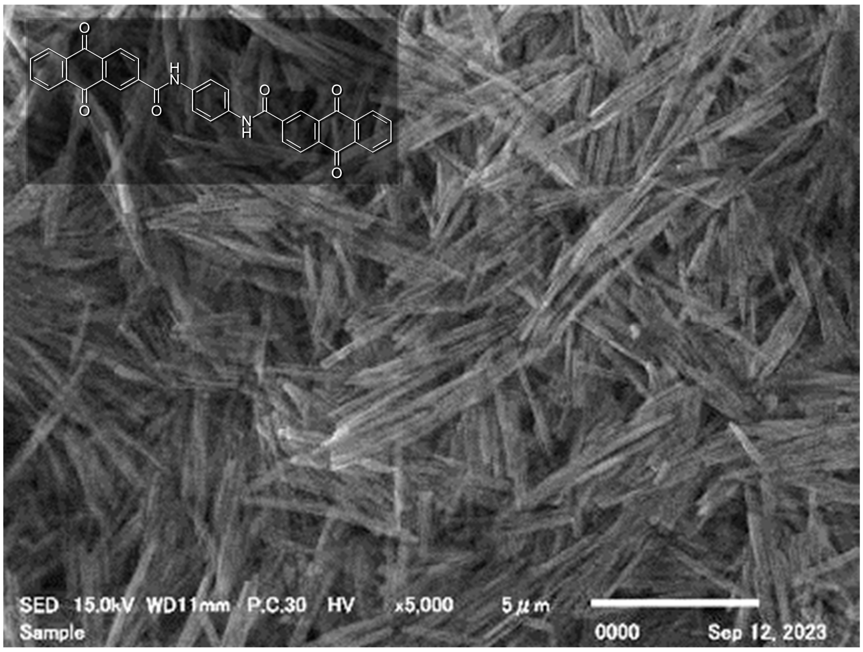

b

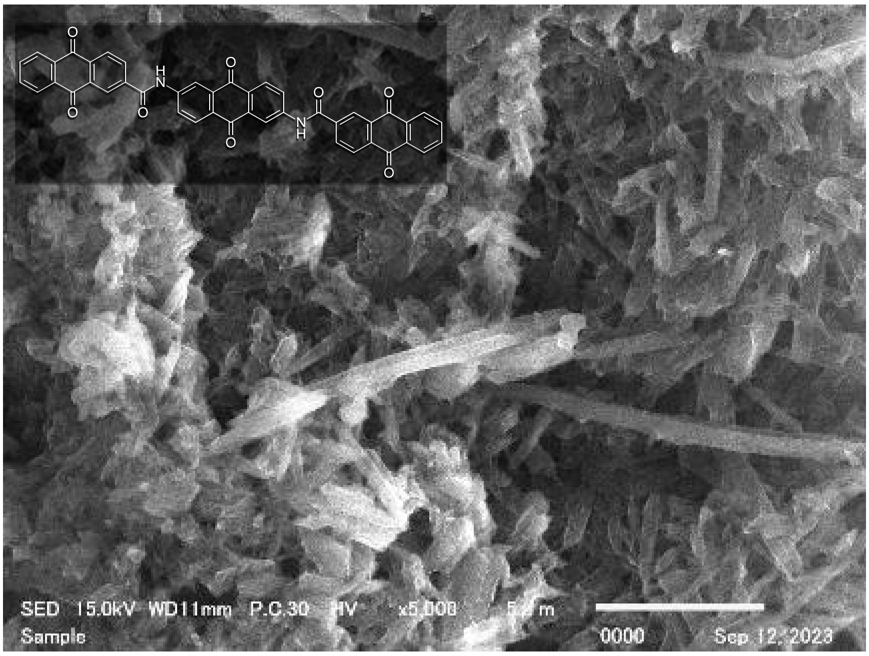

Figure S5. SEM images for (a) the amide-bonded dimer and (b) amide-bonded trimer.

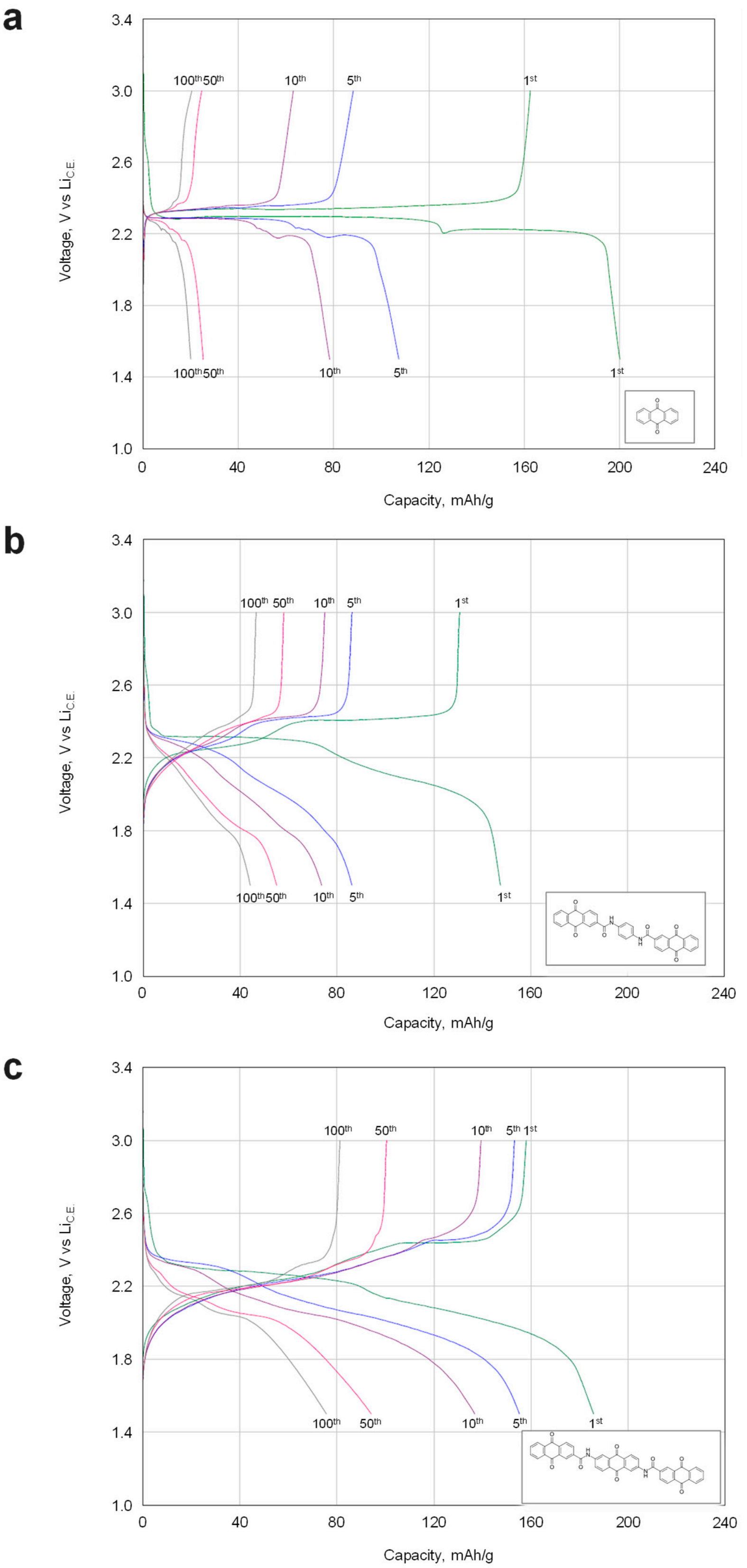

Figure S6. Charge/discharge curves of 1st, 5th, 10th, 50th, and 100th cycles for the electrodes using (a) AQ, (b) AQ amide dimer, and (c) AQ amide trimer at the current density of 40 mA/g.

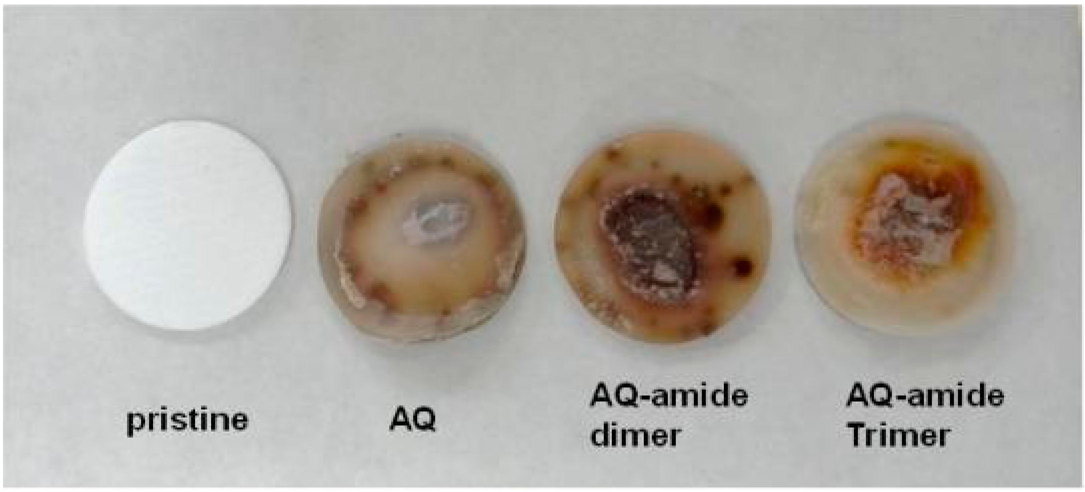

Figure S7. Photo images of the separators after 100 cycles.
